# New Sources for Comparative Social Science: Historical Population Panel Data From East Asia

**DOI:** 10.1007/s13524-015-0397-y

**Published:** 2015-06

**Authors:** Hao Dong, Cameron Campbell, Satomi Kurosu, Wenshan Yang, James Z. Lee

**Affiliations:** 1 Division of Social Science, The Hong Kong University of Science and Technology, Clear Water Bay, Kowloon, Hong Kong, China; 2 Division of Social Science, The Hong Kong University of Science and Technology, Hong Kong, China; 3 Faculty of Foreign Studies, Reitaku University, Kashiwa, Japan; 4 Institute of Sociology, Academia Sinica, Taipei, Taiwan; 5 School of Humanities and Social Science, The Hong Kong University of Science and Technology, Hong Kong, China

**Keywords:** Longitudinal data, Historical demography, East Asia, Population registers, Comparison

## Abstract

Comparison and comparability lie at the heart of any comparative social science. Still, precise comparison is virtually impossible without using similar methods and similar data. In recent decades, social demographers, historians, and economic historians have compiled and made available a large number of micro-level data sets of historical populations for North America and Europe. Studies using these data have already made important contributions to many academic disciplines. In a similar spirit, we introduce five new microlevel historical panel data sets from East Asia, including the China Multi-Generational Panel Dataset–Liaoning (CMGPD-LN) 1749–1909, the China Multi-Generational Panel Dataset– Shuangcheng (CMGPD-SC) 1866–1913, the Japanese *Ninbetsu-Aratame-Cho* Population Register Database–Shimomoriya and Niita (NAC-SN) 1716–1870, the Korea Multi-Generational Panel Dataset–Tansung (KMGPD-TS) 1678–1888, and the Colonial Taiwan Household Registration Database (CTHRD) 1906–1945. These data sets in total contain more than 3.7 million linked observations of 610,000 individuals and are the first such Asian data to be made available online or by application. We discuss the key features and historical institutions that originally collected these data; the subsequent processes by which the data were reconstructed into individual-level panels; their particular data limitations and strengths; and their potential for comparative social scientific research.

## Introduction

Comparison and comparability lie at the heart of social science, but precise comparison is virtually impossible without using similar methods and similar data. Comparable historical data sets are especially scarce. Until very recently, this was particularly true for micro-level data on social and demographic behavior in past populations.

In recent decades, however, social demographers, historians, and economic historians have compiled and made available a large number of household- and individual-level data sets describing historical populations in North America and Europe (Ruggles 2014). The creation and release of these large data sets have allowed researchers to move beyond broad comparisons of aggregates and produce comparative insights at the meso- and micro-levels. Prominent examples include the vast collection of historical and contemporary census data available from the Integrated Public Use Microdata Series (IPUMS) and the North Atlantic Population Project (NAPP), as well as other Western historical data projects, such as the BALSAC Population Database, the Historical Sample of the Netherlands (HSN), Le Programme de Recherche en Démographie Historique (PRDH), the Scanian Economic Demographic Database (SEDD), the Umea Demographic Database (UDDB), and the Utah Population Database (UPDB).^1^ These advances in historical population data construction have contributed enormously to the development of comparative historical demography in particular, and comparative social science in general.^2^

In this article, we introduce five household- and individual-level historical panel data sets for East Asian populations that are similar enough in content and organization to be compared not only with each other, but also with their European and North American counterparts. These data sets include the China Multi-Generational Panel Dataset – Liaoning (CMGPD-LN) 1749–1909, the China Multi-Generational Panel Dataset – Shuangcheng (CMGPD-SC) 1866–1913, the Japanese *Ninbetsu-Aratame-Cho* Population Register Database – Shimomoriya and Niita (NAC-SN) 1716–1870, the Korea Multi-Generational Panel Dataset – Tansung (KMGPD-TS) 1678–1888, and the Colonial Taiwan Household Registration Database (CTHRD) 1906–1945. Altogether, these five data sets contain 3.7 million linked observations of 610,000 individuals, with more individuals and observations to come.

We divide this article into six parts. First, we summarize early efforts to produce systematic comparable data at the national, regional, household, and individual levels and their academic contributions. Next, we discuss the key features of these new East Asian data. The following two parts introduce the historical institutions that produced the original data and the subsequent processes by which these data were transcribed and reconstructed into individual-level panels. In the concluding two parts, we review the strengths and limitations of these data as well as their potential for social science inquiry.

## Development of Data Comparisons

Large-scale cross-national comparative social science emerged in the mid-twentieth century with the creation and dissemination of increasingly detailed and systematic data sets, initially at the macro-level, and then at the meso- and micro-levels. The first such global comparative enterprise may well be the Human Relation Area Files, which beginning in 1949 made a collection of materials on human behavior, culture, and society available to the academic community, first in print and beginning from 1994, and then online (Ember 1997). In the 1960s and 1970s, such quantitative comparative projects as the Princeton European Fertility Project began to make use of meso-level data sets consisting of provincial social and demographic indices. However, it was not until the 1970s and 1980s—when studies began using individual- or family-level data from family reconstitutions and historical censuses— that historical demography emerged as a distinct subfield within population studies, historical sociology, comparative social science, and various subfields of health science. Moreover, it was not until the creation and release of the Integrated Public Use Micro Series (IPUMS) and other micro-level data sets largely beginning in the 1990s that these population-related subfields became a central focus of academic attention (Ruggles 2014).

**Fig. 1:**
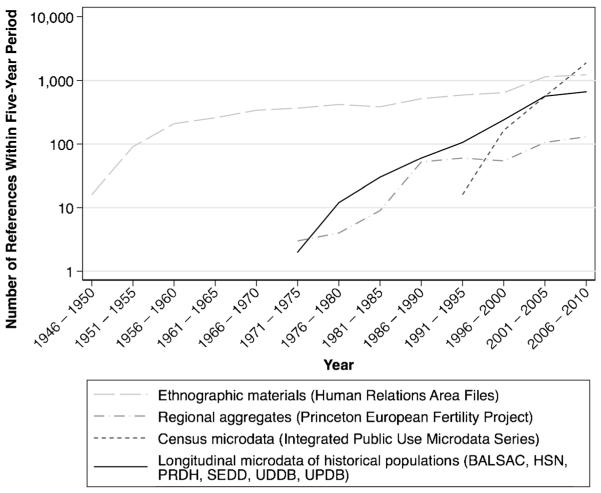
Google Scholar citations generated by comparative “big” social science data

The rapid increase in the numbers of publications referencing these major data sets or projects illustrates the contributions to scholarship of these successive advances in data and associated methods.^3^ Figure 1 provides counts of publications since the middle of the twentieth century by Google Scholar that mention these data sets and/or projects by five-year period. In each case, the availability of systematic comparative data inspired a sustained stream of academic references. The first global data set (the Human Relations Area Files) and the first major historical demographic comparative project (the Princeton European Fertility Project) continue to be referenced by 1,000 and 100 papers, per five-year period, 60 and 40 years, respectively, after they were first created.

Even more striking is the recent, rapid, and still ongoing increase in interest in individual- and family-level microdata (Ruggles 2014). IPUMS, in existence for only two decades and until recently consisting solely of historical and contemporary census data from the United States, now generates almost 2,000 references per five-year period.^4^ Finally, and most relevant to the East Asian data sets introduced here, even though the best-known complex longitudinal data sets describing individuals and households have been publically available only for at most two decades, they already generate nearly 700 new academic references per five-year period.

The simultaneous development of advanced statistical methods to analyze complex longitudinal data has allowed quantitative social science to move from comparison of descriptive aggregate statistics to examination of differences within populations, as well as measurement of associations between variables at the individual and family level. This shift has facilitated the development of more ambitious explanatory and causal models that link demographic behavior with current and past context and circumstances. However, these advanced methods demand increasingly complex and detailed data, including not just vital events and family composition but also occupation; socioeconomic status (SES); wealth; and economic conditions, such as food prices. Because the meaning of occupation, SES, and wealth varies across contexts, the data requirements for comparative historical demographic research are increasingly challenging.^5^

Quantitative historical comparison through the application of these new data and methods has evolved from describing regional or national differences to uncovering similarities within differences, and most recently identifying differences within similarities (Lundh et al. 2014). The Princeton European Fertility Project, an early comparative project, tested existing explanations for the European fertility decline based on computation and comparison of national and provincial differences in demographic rates and socioeconomic indices throughout Europe (Coale and Watkins 1986). Although the Princeton project substantially improved our knowledge of the fertility decline, the aggregate rates and indices on which it relied did not allow for micro-level comparisons that link demographic behavior to individual or household context.

The Eurasian Project in Population and Family History is one recent example of comparative historical demography that uses micro-level data to identify similarities within differences at opposite ends of Europe and Asia in the past.^6^ It makes use of individual-level longitudinal data from historical household registers to compare sociodemographic behavior in a variety of communities in southern Sweden, eastern Belgium, northern Italy, northeastern Japan, and northeastern China. These comparisons allow us to relate demographic behavior to individual, household, and community contexts across Eurasia.^7^ The overall conclusions, summarized in three volumes published as the MIT Press Eurasian Population and Family History Series, focus on East-West divergence and convergence, and challenge current macro historical sociological theories without, however, proposing alternatives (Bengtsson et al. 2004; Lundh et al. 2014; Tsuya et al. 2010). The results suggest that before we attempt to produce new grand social theories at a global scale, we need first to make more detailed comparisons within East and West, focusing on communities that have similarities in terms of background and context.

Recognition of limitations to the focus on East-West comparison in the Eurasian Population and Family History Project inspire our new effort to map similarities and differences in East Asian population behavior through comparative analysis of population register databases: the East Asian Population and Family History Project. To distinguish from the earlier Eurasian Population and Family History Project (EAP I), we call this new project EAP II, which focuses specifically on neighboring populations in East Asia that are more similar in terms of background and context. Participants have already met three or more times at EAP II–related meetings, and have met less formally in other venues. These meetings have already yielded a collection of papers on migration in historical East Asia (Campbell 2013; Kim et al. 2013; Kye and Park 2013; Son and Lee 2013; Tsuya and Kurosu 2013). The coordination and cooperation that we hope to promote in EAP II will be the first step to such detailed comparison.^8^

## New East Asian Microdata

**Map 1:**
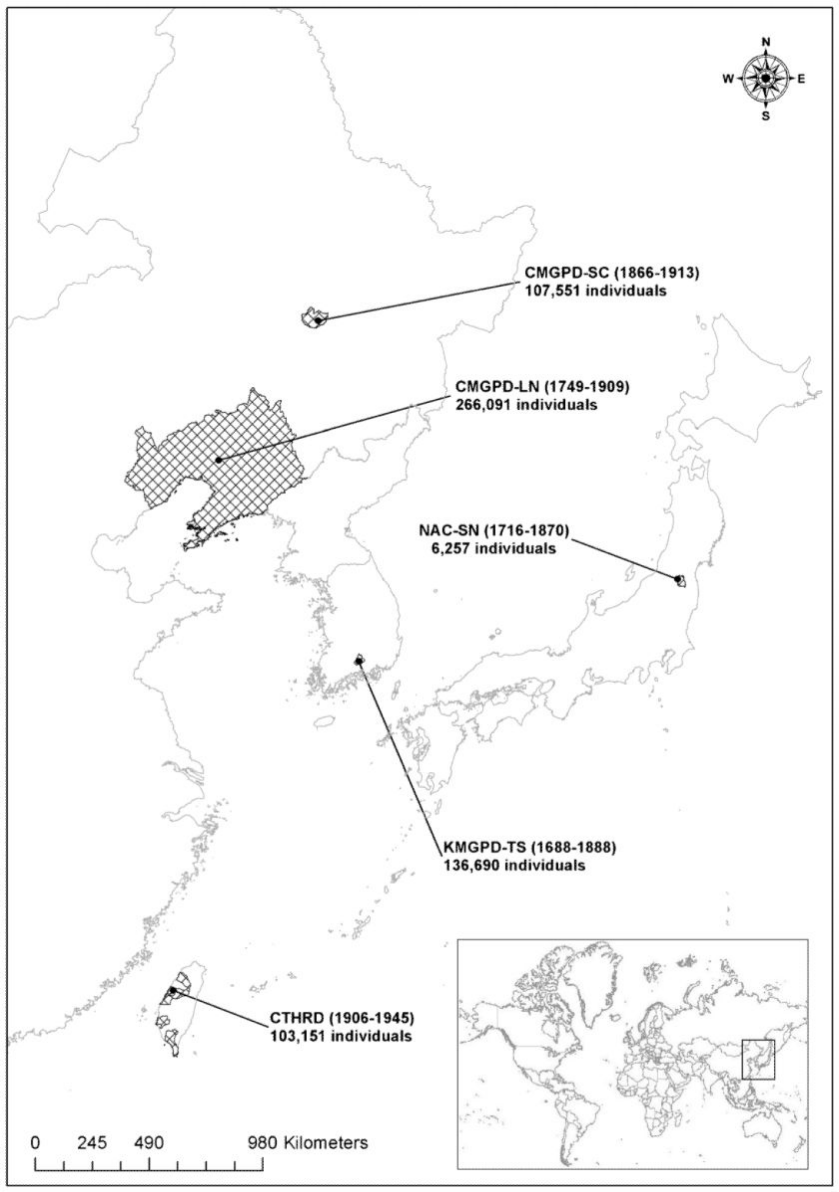
EAP II study populations

The five EAP II data sets are from four distinct regions in East Asia, identified on Map 1, scattered over an area of less than 4 million square km, measuring 1,700 km east to west and 2,300 km from north to south. These regions are geographically contiguous and share similar though far from identical social structures, cultural norms, and political institutions and ideologies.

All five EAP II historical panel data sets are longitudinal in the sense that they contain linked records for individuals over time. Longitudinal data on individuals are valuable because they allow present behavior to be linked with prior circumstances. This allows researchers not only to describe patterns of behavior but also to explain their causes and consequences. Unlike aggregate-level time series that reflect only national, regional, or community averages, individual-level longitudinal data provide life histories for each individual, which makes possible disentangling the complicated relationships between individual behaviors at different time points.

These East Asian data sets, like the EAP I and most European historical panel data sets, are not nationally representative.^9^ Each covers only a limited number of communities; however, unlike proportionally representative samples, they do so in their entirety. They are historical analogs to the contemporary data collected around the world by the participants in the International Network for the Demographic Evaluation of Populations and their Health (INDEPTH) Network (Sankoh and Byass 2012). 10 These data include information on vital events—such as fertility, mortality, marriage, migration, and longitudinal information—on household context and individual characteristics for all individuals in their respective registration areas. The EAP II data sets also record such details as occupation, kinship, (usually) property, and (sometimes) civil service examination attainment, which allow us to aggregate dynamic information on community and household context based on individual information. These data are usually constructed from household or civil registers that survive to the present day. Such historical sources were originally compiled by local governments in connection with population regulation, taxation, religious investigation, and other administrative functions (Ding et al. 2004; Hayami 1979; Kurosu 2002; Lee et al. 2010; Son 2007).

The five EAP II data sets are accessible online or in person subject to application. The CMGPD-LN and the CMGPD-SC and associated documentation are available from an Inter-University Consortium for Political and Social Research website.^11^ The digital images and files for the KMGPD-TS, the Tansung household registers (THR), are also available online,^12^ as are longitudinal links that connect individuals across registers.^13^ The CTHRD is maintained by the Program for Historical Demography (PHD) at the Academia Sinica in Taipei. Researchers can apply for access to data through the PHD website. 14 The NAC-SN was originally constructed by Akira Hayami and his colleagues in Japan, and is now housed at the Population and Family History Project at Reitaku University. At present, researchers may submit a proposal to the Population and Family History Project at Reitaku University, and if approved, carry out the analysis at Reitaku University.^15^

Additional data sets constructed from East Asian household registers exist and may eventually be made available. The China Multi-Generational Panel Dataset–Imperial Lineage (CMGPD-IL) describes 120,000 individuals over 13 generations who belonged to the Qing Imperial Lineage from 1616 to 1936 and is already entered and linked in its entirety (Lee et al. 1993). Choson dynasty Korean household registers from Daegu County and Jeju Island, similar to the original sources for the KMGPD-TS, have also been digitized, along with available data of urban population in Seoul transcribed from colonial household registers under Japanese government. Although the data derived from Japanese population registers presented here include only two villages, data for another three villages and one local town in the same region are already entered, and additional data from other regions in Japan are being entered.^16^ The complete CTHRD includes 14 other locations that are also available for access through the PHD website. More locations in Taiwan are being added.

The EAP II data sets provide comparable and detailed information on demographic and socioeconomic characteristics of individuals and households. Table 1 summarizes their contents. They all provide the age of individuals^17^ and annotate the recent occurrence of key demographic events, such as birth, death, marriage, and migration. Additionally, they all specify individual’s relationship to the household head, record the village of residence, and include information on occupation of males or at least household heads. The CMGPD-LN and CMGPD-SC record occupation only for state employees, such as local officials, artisans, and soldiers. The CTHRD, KMGPD-TS, and NAC-SN record other occupations, including servant and merchant. They all contain at least some information on physical disability and disease, but such information is less consistent, systematic, and comparable. The CMGPD-LN, CMGPD-SC, and KMGPD-TS indicate titles associated with the civil examination system, which are indicative of a high level of education. The CMGPD-SC, NAC-SN, and CTHRD also have information on household landholding.^18^ Most importantly, all the data sets allow for longitudinal linkage of individuals and linkage of individuals to their various coresident and noncoresident kin,^19^ which we describe in the upcoming section, Data Construction.

All these registers provide information that can be used to categorize individuals or households according to their social and economic status: occupational prestige for the CMGPD-LN; property entitlements for the CMGPD-SC; social status for the KMGPD-TS; household head’s occupation and property tax for the CTHRD; and a combination of social status and tax liability for the NAC-SN. These measures are not directly comparable across populations. Previous studies have used them, however, to categorize individual SES into high/middle/low and then examine gradients in demographic behavior. Analyses of the CMGPD-LN typically differentiate individuals according to the status of the state farm population with which they were affiliated and the official position they held, if any. Comparatively, analyses of the CMGPD-SC population usually divide the population into three categories according to their property entitlement: 64.4, 34, and 0 hectares (Chen 2009). The KMGPD-TS recognizes three broad categories in the original data: nobles (*yangban*), commoners (*sangmin*), and subordinates (*nobi*). The CTHRD allows for households to be differentiated by occupation of household head or taxed household landholding: high SES refers to households who paid more than 50 *yuan* (in colonial Taiwan currency) in land tax or the heads who had such white-collar professional occupations as administrative officials, doctors, teachers, or other professionals; middle SES refers to households who paid 1 to 49 *yuan* in land tax or to heads who had regular blue-collar or retail jobs; low SES refers to households who paid less than 1 *yuan* in land tax or to heads who were itinerant peddlers or heavy laborers (Hsieh and Chuang 2005). The NAC-SN divides households according to both principles. It records formal statuses, differentiating titled peasants (*honbyakusho*) who owned land from landless peasants (*mizunomi*). It also records household tax liability for titled peasants, who were assessed based on the productivity of their land, regardless of their formal status.

## East Asian Household Registers and Historical Institutions

The registers from which these data sets were created are products of historical systems of civil, financial, and military administration. The CMGPD-LN and CMGPD-SC are transcribed from triennial and annual Eight Banner population registers, respectively, from Liaoning province between 1749 and 1909 and from Shuangcheng County in Heilongjiang province between 1866 and 1913, in northeast China.^20^ The Eight Banner system was a civil and military administrative system organized by the Qing to govern the Manchurian and Mongolian provinces in Greater North and Northeast China, as well as the Qing garrison populations in China Proper.^21^ The vast majority of the population in the CMGPD-LN were descendants of Han Chinese migrants who migrated from Shanxi, Hebei, and Shandong province to Liaoning after the founding of the Qing dynasty. There were also a small number of indigenous and descendants of earlier settlers who according to their surname or their registered status were Mongol, Manchu, or Korean. The CMGPD-SC population consisted of the descendants of migrants who arrived in Shuangcheng in the early nineteenth century. The original migrants were drawn from Eight Banner populations in Beijing and elsewhere in northeast China. According to the registered ethnicities recorded in the registers, they were a mixture of Manchu, Han, Mongol, and other groups.

The CMGPD-LN and CMGPD-SC registers are organized first by the administrative affiliation of the population, and then within register, by village of residence, household group, and household. Within households, individuals are listed according to their relationship to the head. Administrative affiliation is an important dimension of status and largely hereditary. Families remain affiliated with their original administrative population even after they move elsewhere in the region; and in the case of the CMGPD-LN, continue to be recorded in their original registers, although with their new location identified. Two households containing related individuals may be listed next to each other in the registers even though they reside in separate villages. As a result, both the CMGPD-LN and CMGPD-SC are valuable sources for migration and community studies because they not only provide the same basic information on households as other data sets but also allow for the tracing of households that move within the region and explicitly annotate individual departure from the region.

With a combined coverage of more than 800 communities across a diverse variety of geographic and socioeconomic contexts, analysis of the CMGPD-LN and CMGPD-SC should continue to produce findings that improve our understanding of general patterns of social and family organization in China, including the spatial dimensions of social organization (Lee et al. 2010; Wang et al. 2013). The physical locations of all the communities in the CMGPD-SC are known with precision, as are the physical locations of the 200 or so communities in the CMGPD-LN that accounted for 90 % of the population.

The KMGPD-TS is transcribed from triennial Korean civil household registers (*hojŏk*) compiled between 1678 and 1888 from Tansung County in South Korea.^22^ Whereas the registers for the CMGPD-LN and CMGPD-SC record only those individuals affiliated in some way with the Eight Banner administrative and military system itself, the Tansung registers were intended to cover all people who actually resided in the area, without consideration of their political status or identity.^23^ The population consisted of largely peasants but also local nobles and servile households or subhouseholds (*nobi*).^24^ In the register, each individual was assigned to a household (*ho*). Then, households were organized into *tong* (five-household units), *ri* (village), and *myeon* (subcounty) in ascending order. The Tansung registers covered eight *myeon*, each in a separate register series. Because inclusion was based on administrative jurisdiction of residence, the Tansung registers sought only to record people who actually reside in Tansung. Unlike the CMGPD-LN, they do not follow individuals who leave the area except to indicate that they have left. Sometimes they specify destination.

The NAC-SN is transcribed from a set of Japanese population registers (*ninbetsuaratame-cho*) from two villages, Shimomoriya and Niita, in northeast Japan between 1716 and 1870.^25^ Each year, usually around lunar March, officials registered the residents in these villages and recorded any vital event that the individual experienced in the preceding year. Residents of the two villages were mostly peasants. Like the Tansung registers, the NAC registers also record individuals based on their administrative jurisdiction of residence. Although it is impossible to follow individuals after they leave the village, the registers record the year in which they left, and always record their reason for departure and their destination.

The CTHRD is transcribed from Taiwan household registers (*hujiziliao*) compiled by the Japanese colonial administration from 1906 to 1945.^26^ The sample analyzed here covers eight locations in north and central Taiwan.^27^ This sample includes some urban areas, but the majority of the population recorded in the Taiwan colonial household registers were farmers. In contrast with the annual or triennial CMGPD-LN, CMGPD-SC, KMGPD-TS, and NAC-SN, the colonial Taiwan registers, like the eastern Belgian registers in the EAP I, were updated continuously as vital events and other information occurred. Each household in the original register had one or more pages according to the household size, and each household member was represented by a column on that page in which their vital events and other information were recorded.^28^ If changes occurred that fundamentally altered the household—for example, the household head was replaced—the original page would be crossed out, and a fresh entry started on a new page with a new household head. Although the Taiwan colonial registers do not follow individuals who moved out of the community or trace those who moved in, they provide information on the time of the move as well as the destination or origin. Importantly, the information on timing allows for the censoring of observations in event-history analysis of demographic behavior.

## Data Construction

All five types of household registers require linkage of entries for the same individual in different locations to produce life histories that can be subjected to longitudinal analysis. Although their content resembles that of the large longitudinal databases of historical Western populations being constructed from linked parish and tax data (Mandemakers and Dillon 2004), the organization and format of the original data differs fundamentally, requiring a distinct approach to data set construction. The original registers from which the CMGPD-LN, CMGPD-SC, KMGPD-TS, and NAC-SC were constructed resemble annual or triennial censuses in the sense that they provide detailed snapshots of the population at fixed intervals in which individuals are observed repeatedly, while the Taiwan colonial registers consisted of one page for each household that was updated as events occurred. The annual and triennial household registers do not trace individuals from one register to the next, and they require manual or automated longitudinal linkage to produce the life histories that relate outcomes and behaviors to characteristics and context earlier in the life course. The continuous Taiwan colonial registers also require linkage of information about the same individual recorded in different households at different stages of their life to produce life histories. Although the page for each household offers complete records of events that occur during the period covered by the entry, the same individual may appear in the entries of different households at different periods of their life.

Through linkage, we have transformed these data into historical panel data sets that follow individuals across time and families across generations. In the CMGPD-LN, CMGPD-SC, and KMGPD-TS, we linked observations of the same individual in adjacent registers. Such linkage is straightforward in the CMGPD-LN and CMGPD-SC because households and their members are mostly listed in the same order in each register. Coders carry out linkage at the time they enter the data. In the KMGPD-TS, households do not appear in the same order in adjacent registers. We developed a process in which analytical software made a first pass and proposed candidate links based on name, calculated year of birth, and other information, and then coders adjudicated among the proposed links and created final links of their own.^29^ After longitudinal linkage is complete, the software concatenates information from all the observations of an individual to produce life history information.

The NAC-SN and CTHRD had additional complexities. The transcription of the NAC-SN predated the contemporary era of database software. Individuals were first transcribed manually on time-series data sheets—called Basic Data Sheets (BDS), which were organized by household and then entered into databases. Household and individual histories were constructed based on unique household and individual identifiers (for specifics, see Ono 1993; Tsuya 2007). The transcription of the CTHRD relied on a specially designed data entry program, which allowed for dynamic linking of information about the same individual recorded on different register pages as coders entered data.^30^

**Fig. 2:**
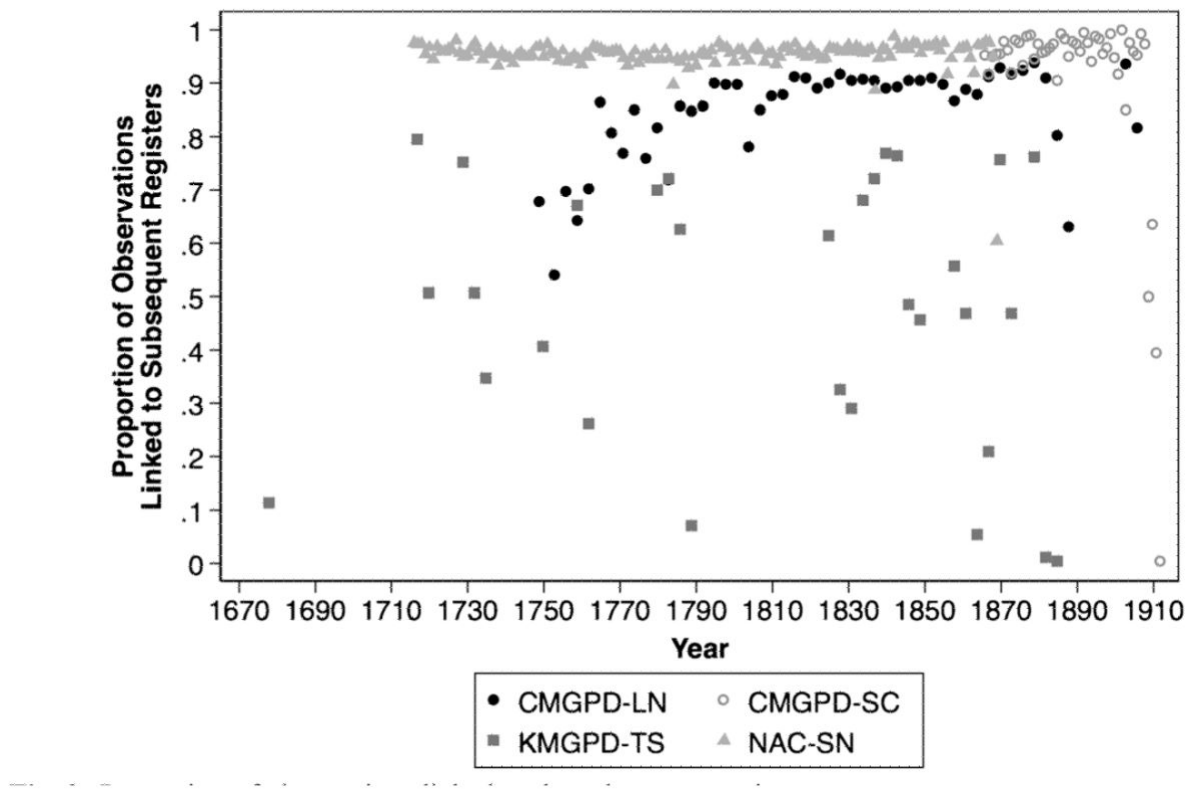
Proportion of observations linked to the subsequent registers

In the CMGPD-LN, CMGPD-SC, KMGPD-TS, and NAC-SN, the proportion of observations linked to an observation of the same individual in a subsequent register is generally high. Figure 2 displays these proportions.^31^ Because out-migration for work, marriage, and even escape are specifically annotated in all five sources,^32^ variations in linkage success across register pairs are largely the product of missing registers. When a register has not survived, links are made between observations in the extent registers. As discussed in detail in the upcoming section, Data Limitations and Implications for Comparability, individuals whose exits or entrances were recorded in the missing original registers appear or disappear with no explanation in the period between the two surviving registers. Longer gaps between surviving registers are associated with higher proportions of individuals whose first or final appearance was in a missing register, and who therefore cannot be linked.

The annual NAC-SN and CMGPD-SC have as high as 90 % to 95 % pairwise linkage rate between registers. In the CMGPD-LN, the overall pairwise linkage rate is approximately 90 %. This percentage is especially high considering that the CMGPD-LN is based on triennial rather than annual registers, and it covers an area of more than 600 villages, which is much larger than the other data sets. In the KMGPD-TS, gaps due to missing registers are longer and more common, reducing pairwise linkage rates to sometimes as low as 2 %. However, if we consider only those linkage rates between surviving registers that are three years apart, they are 70 % to 80 %.

By concatenating the links made between pairs of registers, we reconstruct life histories for individuals. Table 2 presents the distribution of individuals according to years under observation in each data set. In the Years Under Observation column, “1” refers to individuals who are observed in only a single register and are not linked to any adjacent register; and “2–3” refers to individuals who have at least two linked observations, whether annual or triennial. CMGPD-SC has the lowest proportion of individuals who are only observed once (7.11 %), followed by the NAC-SN (13.38 %), CMGPD-LN (15.61 %), CTHRD (31.90 %), and KMGPD-TS (52.36 %). CMGPD-LN has the highest proportion of individuals who can be followed for 22 or more years: 43.57 %. In CMGPD-SC, NAC-SN, and CTHRD, the relevant percentages are 39.80, 34.69 and 23.57, respectively.

Linkage of individuals to their family members is based on the recorded relationship of each individual to his/her household head. When detailed relationship to the household head is recorded, it is possible to use it to identify relationships between any pair of individuals within the same household and link them with each other. Links between parents and children are especially useful because they may be cumulated across generations to reconstruct pedigrees and then identify distant kin, including those residing in other households or even other villages.

The success of such family linkage depends on the precision of the relationships recorded in the register and on whether individuals were ordered in a consistent fashion in the register. The original CMGPD-LN and CMGPD-SC registers always list wives next to their husbands and children next to their parents. They also describe individual relationship to the household head in great detail. In the NAC-SC and CTHRD, the registers also record relationship to household head with great precision so that the completeness of family linkage is comparable with the CMGPD-LN and CMGPD-SC. In contrast, the original registers of the KMGPD-TS list household members of the same generation together without further specification of their relationship to the head. As a result, although it is easy for coders and software to link wives with husbands and children with parents in the CMGPD registers, such linkage in the KMGPD-TS is much more difficult, and indeed, often impossible. For example, in a three-generation household headed by someone in the senior generation, we cannot link children with their parents and grandparents if the generation of the parents or grandparents contains more than one married couple.

Because of the time span, we can iterate through the links of children with parents to identify the distant ancestors of individuals. As shown in Table 3, the CMGPD-LN has the highest proportion of individuals who can be linked back across multiple generations: 85.46 % of individuals can be linked to the previous generation, the highest among all five data sets. Most of the unlinked individuals are in the earliest registers. They are unlinked because their father passed away before the earliest available register and was not recorded in an existing register. The CMGPD-LN is also remarkable for the number of individuals whose ancestry can be linked back for many generations: 55,243 individuals (20.76 %) can be traced back at least six generations: that is, to their great-great-great-grandfather. For individuals born in the late nineteenth or early twentieth century, the proportions are much higher. Even though the CMGPD-SC and CTHRD also have high proportions of children linked with parents, their limited time span means that multigenerational linkage is limited to four generations. Because the NAC-SN includes only two villages, linkage is limited to families that remained in the villages for multiple generations. For those families, multigenerational linkage is relatively successful. In the KMGPD-TS, multigenerational linkage is less successful, mainly because as discussed earlier, child-parent linkage is more difficult.

## Data Limitations and Implications for Comparability

In the annual or triennial registers, individuals whose exit was recorded in a missing register disappear without explanation in the data set. When gaps between surviving registers are large, many individuals may disappear. In the CMGPD-LN, for example, many pre-1789 registers are missing, and all the registers between 1888 and 1903 were lost to fire. In the CMGPD-SC, only a few registers between 1866 and 1913 are missing, but because the Shuangcheng government archive was destroyed in 1865 during a local rebellion, there are no registers before 1866. Missing registers account for 74,420 unannotated individual exits from the CMGPD-LN (27.97 % of all recorded individuals) and 12,489 unannotated individual exits from the CMGPD-SC (11.61 % of all recorded individuals). In the KMGPD-TS, long gaps are especially common. We have consecutive registers for all eight *myeon* for only two short periods, 1729–1735 and 1780–1786. In other years, especially after 1789, many *myeon* are missing registers. As a result, of the 136,690 unique individuals in the KMGPD-TS, 71,823 (52.54 %) disappear with no explanation. The NAC-SN data are by far the most complete. Only a few registers—1720, 1729, 1846, 1850, 1858, and 1864–1867 for Shimomoriya; and 1742, 1758, 1796, and 1857–1858 for Niita—are missing; consequently, only 5.88 % (368) of recorded individuals disappear without annotation.

**Fig. 3:**
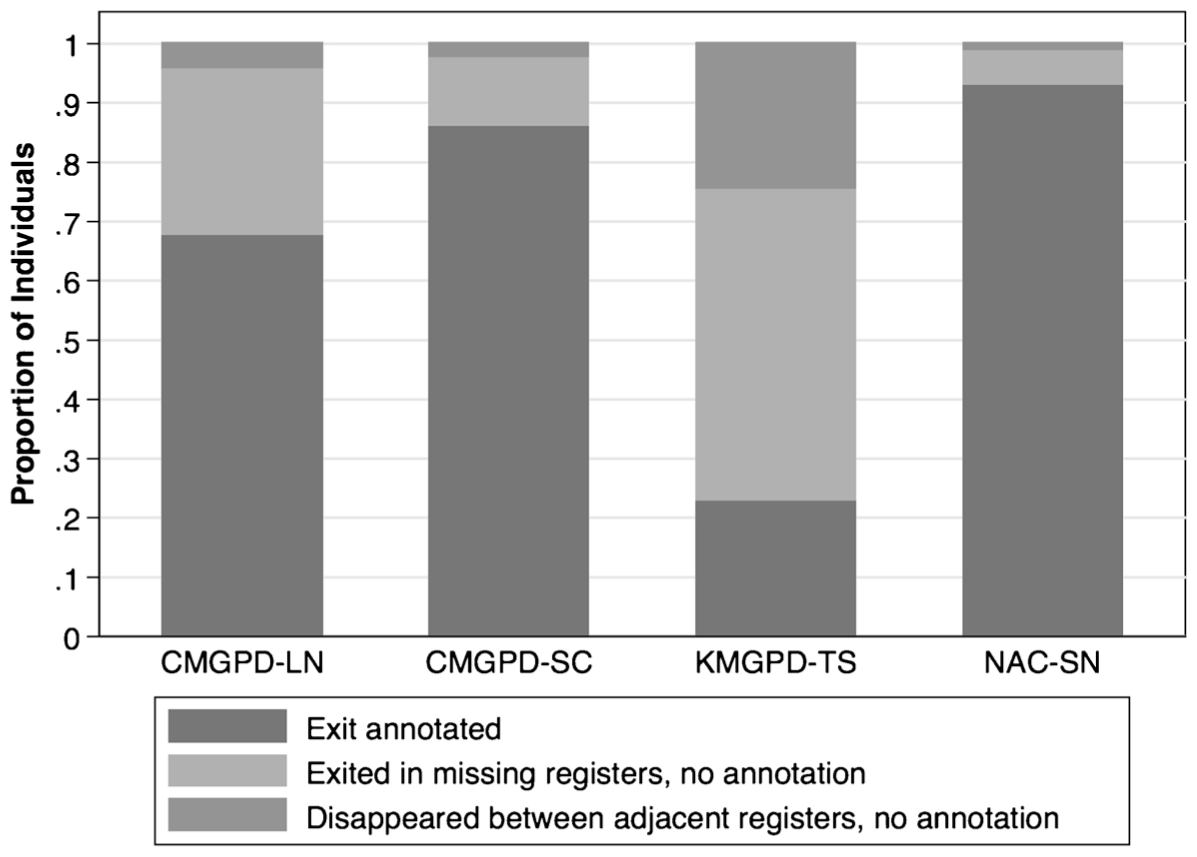
Proportion of individuals without an exit annotation by reasons

Even when there are no gaps between surviving registers, not all exits are annotated. Figure 3 compares the proportions of unexplained disappearances in such circumstances for all five data sets. The problem is the most serious in the KMGPD-TS, where 24.88 % of unannotated exits take place between surviving adjacent registers. According to Kim et al. (2013), this may reflect properties of the registration system. Individuals within the same household tended to disappear together, and the likelihood of unannotated exit was associated with social status. Unannotated disappearances between surviving adjacent registers are much less common in the other data sets. In the CMGPD-LN, CMGPD-SC, and NAC-SC, the proportions of individuals who disappear in such fashion among the total missing individuals are 4.26 %, 2.07 %, and 1.18 %, respectively.

Although disappearances in the CMGPD-LN, CMGPD-SC, KMGPD-TS, and NAC-SN may be dealt with in a straightforward fashion, they are potentially more complex and difficult to deal with in the CTHRD. Because individuals in the annual and triennial data sets are observed at regular intervals, discrete-time event-history analysis may be used, and the observation immediately preceding the disappearance can be excluded. It is not easy to apply similar data restrictions to the CTHRD to address problems caused by disappearances because like traditional family reconstitutions and some European household registers, the CTHRD records only events and transitions. An individual who disappeared may not be distinguished from someone who remained in the household but experienced no additional events that require annotation. That said, disappearances in the CTHRD appear to be very rare. According to Li et al. (2011), at least before 1935, death registration was nearly complete. When individuals migrated out of the community, as noted earlier, the timing of the move and the destination were typically recorded.

**Fig. 4:**
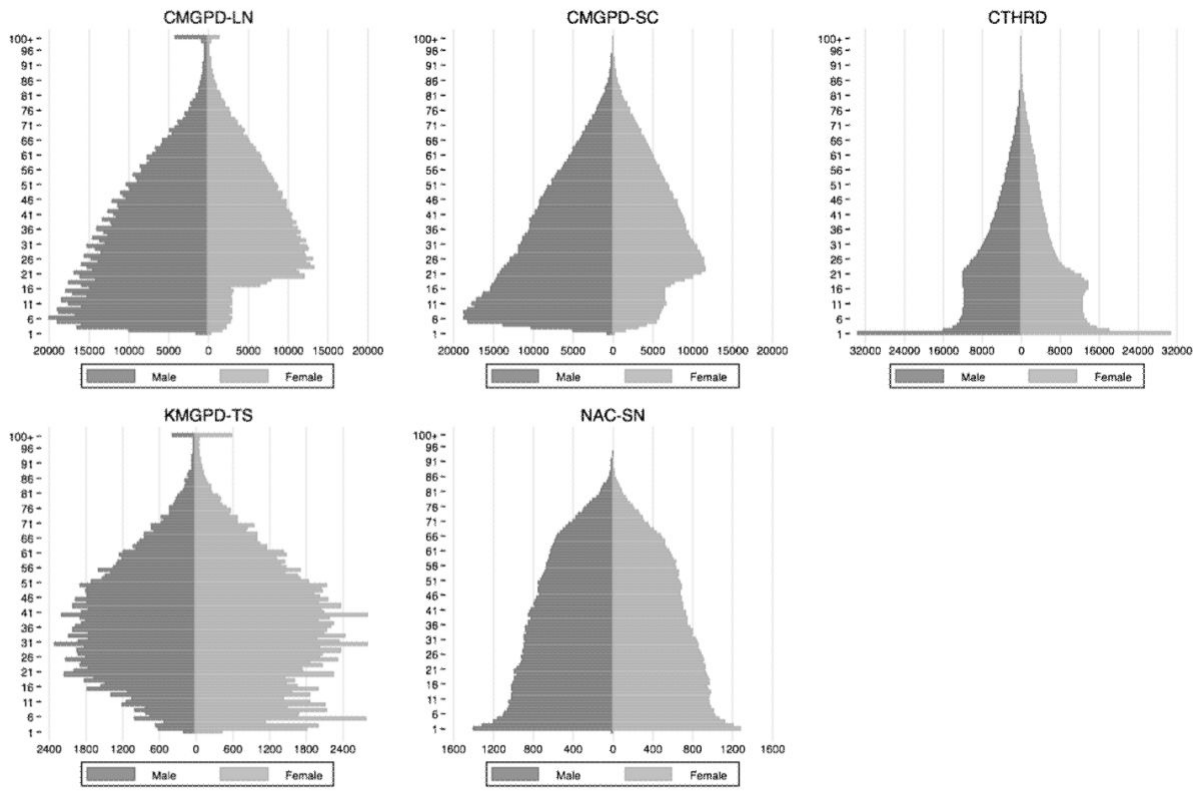
Observation pyramids of the CMGPD-LN, CMGPD-SC, CTHRD, KMGPD-TS, and NAC-SN

Adult males and married and widowed women appear to have been recorded well, but in the CMGPD-LN, CMGPD-SC, and KMGPD-TS, many children were missing. Figure 4, which shows a distribution of ages recorded in the observations, summarizes the basic patterns in recording by sex. In the CMGPD-LN, CMGPD-SC, and KMGPD-TS, parents typically waited until their children were older before they recorded them, and in many cases never recorded children who died in infancy or early childhood. The CMGPD-LN and CMGPD-SC also appear to have omitted many daughters completely, while the KMGPD-TS omits many sons. In the CMGPD-LN, there are six to eight times as many observations of boys as girls. In the CMGPD-SC, there are about three times as many observations of boys as girls. In the KMGPD-TS, however, there are twice as many records of female children as there are for male children. For the CMGPD-LN and CMGPD-SC, the omission of daughters makes the study of assortative mating and female marriage timing difficult. It is difficult to trace wives back to their natal families, and the representativeness of the daughters who are recorded is unclear.

Registration in the CTHRD and NAC-SN appears more complete in the sense that the sex distribution of the recorded populations by is relatively balanced.^33^ The distribution of observations by age shows that the CTHRD, as a continuous record, records births and infant deaths relatively completely; while in such discrete registration systems as the CMGPD-LN, CMGPD-TS, KMGPD-TS, records of new births and infant deaths are relatively incomplete. Records of births and deaths in the NAC-SN are also incomplete, but the problems are much less serious than in the other registers.

**Fig. 5:**
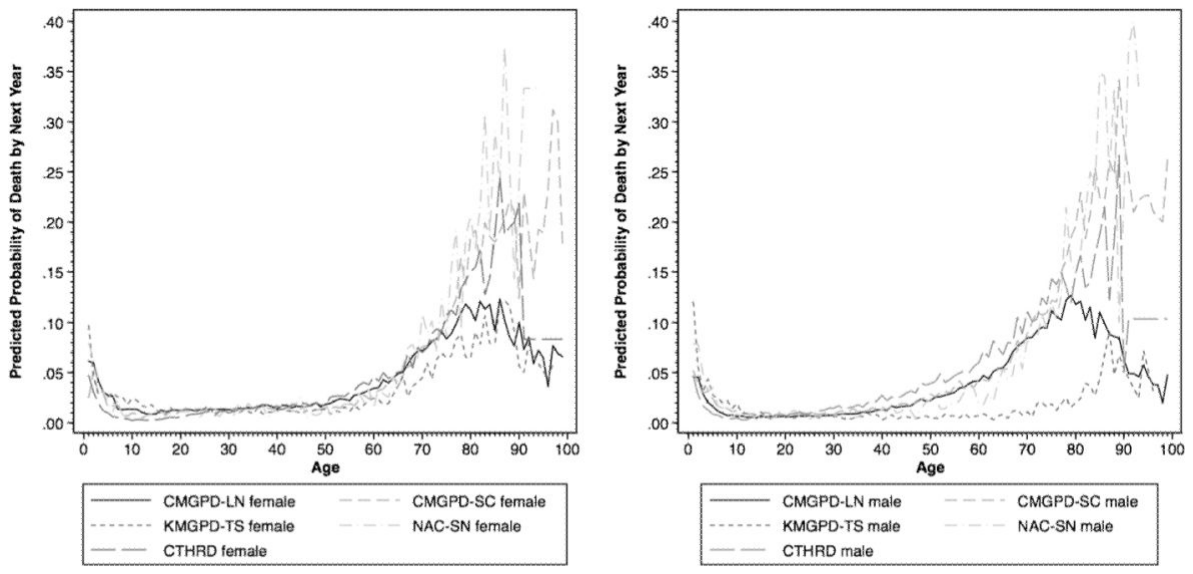
Predicted probability of death by next year by age (left: female; right: male)

The excess of individuals at very advanced ages that is most apparent in CMGPD-LN and KMGPD-TS reflects the failure to record the deaths of a small number of individuals. In both data sets, a small number of people who died did not have their death recorded and were carried forward from one register to the next. Before old age, there are too few cases to be of any significance. In old age, however, as everyone else dies off, these apparent immortals come to dominate the register population. Age-specific mortality rates shown in Fig. 5 confirm that the individuals who appear to have reached very advanced ages had unnaturally low death rates, presumably because they were already dead but were still being carried forward in the registers. Previous studies of mortality using these data (e.g., Campbell and Lee 2005, 2008, 2009; Dong and Lee 2014; Tsuya and Kurosu 2004, 2010) have addressed this by restricting analysis to individuals below age 75.

Meanwhile, Fig. 5 confirms that before old age, age patterns of mortality in these five data sets are broadly consistent with each other.^34^ In other words, the EAP II data should be adequate for comparative analysis of patterns of differential mortality. The consistency of estimates in the CMGPD-LN, CMGPD-SC, NAC-SN, and the transformed person-year CTHRD are especially striking. From very early ages to 75, levels and patterns are similar for both males and females. The KMGPD-TS appears to have the least reliable recording of mortality. Even though estimates for females are in line with those in the other data sets, recorded mortality levels for males appear unusually low.

**Fig. 6:**
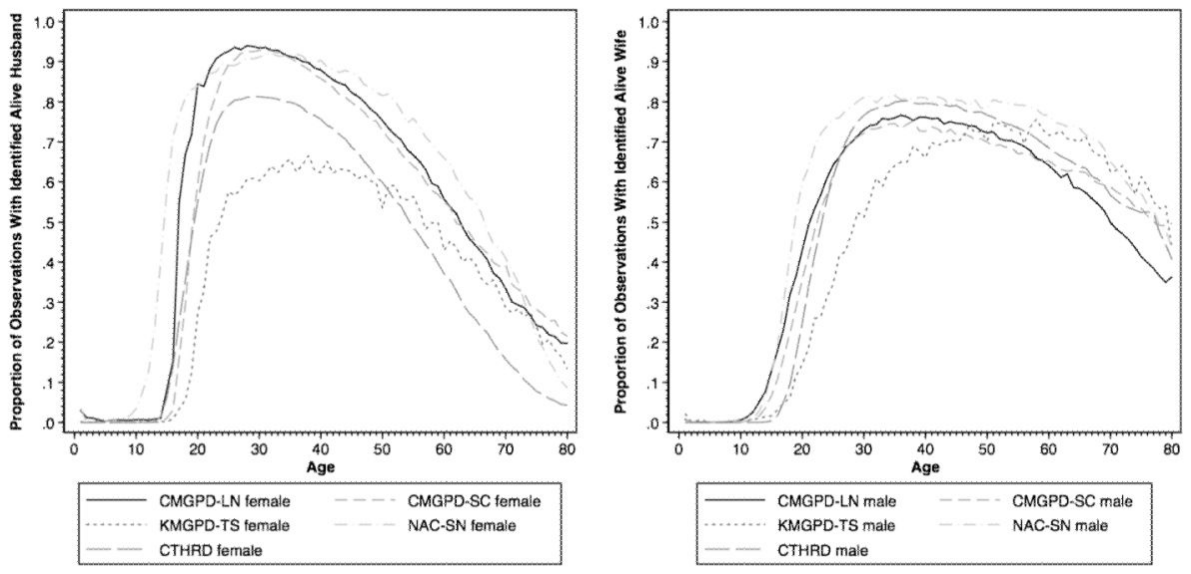
Proportion of observations with identified spouse by age (left: female; right: male)

Marriage also appears to be recorded reliably in most of the data sets. Figure 6 presents the proportions of individuals who can be linked to a spouse. In other words, this measures the proportion of currently married individuals by age. Female marriage was early and universal. In all the studied populations, the proportion of females who are married increases very rapidly in the late teens and early 20s. By age 30, almost all females in these populations are married. In the CMGPD-LN, CMGPD-SC, and NAC-SN, the proportion is much higher than 90 %. The NAC-SN females tend to marry earlier than females in other populations. The proportion of married females identified in the CTHRD data is relatively low. Although the KMGPD-TS has much lower proportions of recorded married females, this is largely a result of the inclusion of *Nobi*, who were less likely to be linked to a spouse. The proportions and patterns of non-*Nobi* females with a linked spouse in the KMGPD-TS, however, look very similar to females in other data sets.

Male marriage was not universal. In every data set except for the KMGPD-TS, men began marrying at around age 15, and 70 % to 80 % were married by the time they were in their late 20s. Males in the NAC-SN married the earliest and in the highest proportions, probably due to their high frequency of remarriage. The KMGPD-TS has the lowest proportions recorded as married for males. Again, we suspect that this reflects underreporting. *Nobi* in particular were especially unlikely to have a spouse recorded, and it is unclear whether this is because they were unmarried, or married but did not report this event.

Additional available information varies across the data sets. The generational depth of the CMGPD-LN, for example, allows for measurement of the characteristics of distant kin. The CMGPD-SC records household landholding, including the location of plots and whether the land was allocated by the state or acquired separately by the household. Both the CMGPD-LN and CMGPD-SC record official position, administrative affiliation, and registered ethnicity. The KMGPD-TS records occupation and social background information for individuals as well as their mothers and fathers. The NAC-SN records many adoptions, as well as detailed information not only on household landholding but also on assets and farming animals of the household. The CTHRD also contains information on female footbinding and smallpox inoculation.^35^

With the information on kinship and residence available in all data sets, we can embed individuals into a conceptual web of two dimensions: one is the relative position within the kin network, and the other is residential location and social position within the community. We can construct measures of community, household, and kinship context by aggregation of characteristics of relevant individuals. We can also construct relative measures that locate individuals within each of these units of organization. With these constructed measures, we can examine how community, kinship networks, and household context interact to shape individual behavior.

Comparison is facilitated by similarities in the cultural background of these locations. These sites are hardly identical, but they do have some features in common that distinguish them from Western populations: most notably, the emphasis on family and kinship as well as Confucian ideology. As suggested by the World Value Surveys (Inglehart and Welzel 2005, 2010), such similarities are still apparent in these societies. Given that the family is of the central importance of social organization in East Asia, individuals’ relationships are a key determinant of their standing in society and also their life chances.

By aggregating information across individuals, we can also construct measures of social and economic status at the level of the household, kin group, and community. All EAP II sources provide some level of detail on social or economic status, allowing for reconstruction of the community and kin group economic and social characteristics. Therefore, in addition to making use of the recorded statuses or occupations of individuals, we can interpret them relative to the social and economic standing of the kin group and the community. This is particularly valuable for research that compares social statuses or occupational groups on individual outcomes between different populations.

## Potential for Comparative Social Science

The five EAP II data sets under discussion have proven useful for historical demography. They should also be valuable for comparative social science in general. Recent usage of similar longitudinal microdata of historical populations for Western populations shows the increasing importance of such records for social scientists not only in population studies, but also in health, economics, sociology, ecology, and other fields.

Ideally, micro-level historical data would combine longitudinal depth with spatial breadth. However, this ideal is rarely achieved because of the limited geographic coverage or survivorship of such historical sources.^36^ Although census data such as IPUMS and the NAPP provide the broadest possible spatial coverage, longitudinal linkage to follow individuals across successive censuses is promising but still difficult.^37^ In contrast, data transcribed from genealogy, parish register, or household register provide relatively complete longitudinal information on individuals and families, sometimes across many generations, but are available only for specific descent groups or communities and are not necessarily representative of their regional context, let alone national context.

The EAP II data are especially noteworthy because they combine longitudinal depth with geographic breath, provide the same information as the best Western longitudinal micro historical data sets, and added together roughly equal the population size of their European counterparts. All together, the five EAP II data sets cover four to eight generations residing in well over 1,000 villages or communities, with a total population size of 600,000 individuals, which is comparable with the combined population of the Scanian Economic Demographic Database (104,000 individuals); the Historical Sample of the Netherlands (78,000 individuals); the Italian Historical Population Database (17,000 individuals); the Umea Demographic Data Base POPUM (around 365,000 individuals); and the most newly released historical European intergenerational longitudinal population, the TRA database, which records 81,000 individuals. In fact, if we consider only linked life histories that allow for longitudinal/panel analysis at the individual level, the EAP II data contain even more available data than these five European data sets.

The five EAP II data sets all derive from common systems of household registration, and can potentially be standardized into identically formatted files and subjected to the same methods of analysis. In principle, it should be possible to combine these East Asian population data into a single large file, which would allow for comparisons of estimated coefficients of different populations within one statistical model rather than on statistical associations from a series of identical models on different data sets separately.

Overall, these East Asian data generate possibilities for important new comparisons on a continental scale. Although they are comparable within East Asia, they may be even more valuable as a basis for comparison with data from other societies and periods. They not only will improve our knowledge of populations in the past but also will contribute new insights into the processes that characterize contemporary populations. All such comparisons, facilitated by recent developments in micro-level “big” social science data worldwide will ultimately lead us to a better understanding of human agency and behavior in general.

## Figures and Tables

**Table 1:** Available information in the five EAP II data sets

	CMGPD-LN	CMGPD-SC	KMGPD-TS	NAC-SN	CTHRD
Data Set Information					
Period	1749–1909	1866–1913	1678–1888	1716–1870	1906–1945
Frequency of update	Triennial	Annual^a^	Triennial	Annual	Continuous
No. of observations	1,513,357	1,346,826	275,042	118,879	481,383
No. of individuals	266,091	107,551	136,690	6,257	103,151
Demographic Information					
Sex	Recorded	Recorded	Recorded	Recorded	Recorded
Age^b^	Yes	Inferred by birthdate	Yes	Yes	Inferred by birthdate
Timing of birth	Year-Month-Date-Hour^c^	Inferred^c^	Year	Year-Month	Year-Month-Date
Physical disability & disease^d^	Males	Males	Males and females	Males and females	Males and females
Timing of death	Three-year period	Year	Three-year period	Year-Month	Year-Month-Date
Marriage	Recorded	Recorded	Recorded	Recorded	Recorded
Residential location	Village	Village	Village	Village	Village
Migration^e^	Tracked within the area	Entrance and exits	Entrance and exits	Entrance and exits	Entrance and exits
Timing of migration	Three-year period	Year	Year	Year	Year-Month-Date
Socioeconomic Information					
Relationship to household head	Yes^f^	Yes	Yes	Yes	Yes
Administrative status^g^	Regular/special duty/servile	Metropolitan/rural/ floating	Yangban/sangmin/ nobi	Honbyakusho/ mizunomi	No
Occupation	Males	Males	Males	Males	Household heads
Civil service examination titles	Males	Males	Males	No	No
Household landholding	No	Yes	No	Yes	Partial

a Although the major part of Shuangcheng Settler—Metropolitan and Rural bannerman—registers are compiled annually, a small set of Shuangcheng floating labor bannerman registers are compiled triennially, which in total accounts for 10 % of observations in CMGPD-SC.

b Ages are calculated by *sui* (Chinese)/*sai* (Japanese)/*se* (Korean), a traditional way to calculate age in East Asia. A person is aged 1 *sui/sai/se* at birth and is one year older after each lunar new year.

c In Shuangcheng, year of birth is calculated from recorded age. In Liaoning, birthdate is recorded reliably only in early registers.

d Information on physical disability and disease is not systematic in the whole recorded population and only of certain limited types.

e In CMGPD-LN, we can continuously observe individuals before and after their legal migration. In the other data sets, we only have information of individuals before out-migration, either legal or illegal. As a result, our observation on such migrants ends after they migrate out, although sometimes their planned destinations are reported in the last records.

f From the year 1789, information on individual’s relationship to household head is available in CMGPD-LN. Before 1789, only information on individual's relationship to head of lineage is available.

g In the CMGPD-LN, such population categories are based on separate sets of population registers that reflect differences in social and political status and entitlement rights. In the CMGPD-SC, although they are all rural residents, metropolitan banner population refers to those immigrants originally from Beijing and Rehe, who are eligible for the highest amount of land allocated by the Shuangcheng government; rural banner population refers to those immigrants from Liaoning who are officially allocated a lesser amount of land and supposed to work as tenants for metropolitan bannerman; floating banner population has no right to claim the ownership of official lands as they migrate to Shuangcheng by themselves rather than commanded by government. In the KMGPD-TS, *Yangban* population refers to the high-level noble population in Korean society; *Sangmin* population refers to middle-status commoner population in the society; *Nobi* population refers to the low-status servile population. In NAC-SN, the status is in general based on the land-tax system. In addition to such major social categories as titled peasants (*honbyakusho*) and tenant peasants without landholding (*mizunomi*), there are other categories such as hereditary servants (*nago*), housing renters (*tanagari*), and Buddhist temple, Shinto shrine, mountain ascetic (*yamabushi/shugen*). In CTHRD, such category is a product of grouping specific occupation of household head and amount of household land tax.

**Table 2:** Individuals by number of years of observation

	CMGPD-LN			CMGPD-SC			KMGPD-TS			NAC-SN			CTHRD		
Years Under Observation	Freq.	%	Cum.	Freq.	%	Cum.	Freq.	%	Cum.	Freq.	%	Cum.	Freq.	%	Cum.
22+	115,948	43.57	43.57	40,824	37.77	37.79	9,845	7.21	7.21	2,167	34.69	34.60	24,321	23.57	23.58
19–21	11,738	4.41	47.99	6,435	5.95	43.74	1,856	1.36	8.57	244	3.90	38.50	6,138	5.95	29.53
16–18	7,768	2.92	50.91	8,151	7.55	51.29	3,980	2.91	11.48	300	4.80	43.30	5,610	5.44	34.97
13–15	8,140	3.06	53.96	7,086	6.57	57.86	3,724	2.72	14.20	349	5.59	48.89	4,181	4.05	39.02
10–12	8,979	3.37	57.34	8,915	8.25	66.11	5,100	3.73	17.93	363	5.81	54.70	4,248	4.12	43.14
7–9	8,390	3.15	60.49	7,793	7.21	73.32	7,661	5.60	23.53	365	5.83	60.53	4,870	4.72	47.86
4–6	38,861	14.60	75.10	10,530	9.75	83.07	6,632	4.85	28.38	469	7.50	68.03	6,358	6.16	54.02
2–3	24,727	9.29	84.39	10,550	9.77	92.84	26,322	19.26	47.64	1,163	18.59	86.62	14,520	14.08	68.10
1	41,540	15.61	100.00	7,736	7.16	100.00	71,570	52.36	100.00	837	13.38	100.00	32,905	31.90	100.00
Total Individuals	266,091			108,020			136,690			6,257			103,151		

**Table 3:** Number of individuals by linked previous generations

	CMGPD-LN			CMGPD-SC			KMGPD-TS			NAC-SN			CTHRD		
Generations	Freq.	%	Cum.	Freq.	%	Cum.	Freq.	%	Cum.	Freq.	%	Cum.	Freq.	%	Cum.
6+	55,243	20.76	20.76	449	0.42	0.43	1,043	0.77	0.76	487	7.78	7.78	** — **	** — **	** — **
5	40,673	15.29	36.05	922	0.85	1.28	1,457	1.07	1.83	414	6.62	14.40	353	0.34	0.34
4	44,923	16.88	52.93	10,396	9.62	10.90	4,090	2.99	4.82	564	9.01	23.41	6,865	6.66	7.00
3	45,206	16.99	69.92	32,135	29.75	40.65	10,012	7.32	12.14	832	13.30	36.71	26,939	26.12	33.11
2	41,345	15.54	85.46	37,368	34.59	75.24	27,443	20.08	32.22	1075	17.18	53.89	31,314	30.36	63.47
1	38,701	14.54	100.00	26,750	24.76	100.00	92,645	67.78	100.00	2885	46.11	100.00	37,680	36.53	100.00
Total Individuals	266,091			108,020			136,690			6,257			103,151		

**Table 4:** Characteristics of EAP II and major Western large-scale micro-level historical demographic data

	Individual Vital Events	Individual SES	Kinship/ Relationship Between Individuals	Intergenerational Linkage	Household Composition Recorded Continuously	Family/ Household SES	Complete Community Coverage
CMGPD- LN	Yes	Yes	Yes	Yes	Yes	No	Yes
CMGPD- SC	Yes	Yes	Yes	Yes	Yes	Yes	Yes
CTHRD	Yes	Yes	Yes	Yes	Yes	No	Yes
KMGPD- TS	Yes	Yes	Yes	Yes	Yes	Yes	Yes
NAC-SN	Yes	Yes	Yes	Yes	Yes	Yes	Yes
BALSAC	Yes	Yes	Yes	Yes	No	No	Yes
IPUMS- USA Linked	Yes	Yes	Yes	No	No	Yes	No
HSN	Yes	Yes	Yes	No	Yes	No	No
SEDD	Yes	Yes	Yes	Yes	Yes	Yes	Yes
UPDB	Yes	Yes	Yes	Yes	No	Yes	Yes

## Footnotes

1 The Handbook of International Historical Microdata for Population Research (Hall et al. 2000) provides a detailed survey of such available historical micro-level population data in the West. Many Western longitudinal micro historical data projects are now affiliated with the European Historical Population Samples Network (EHPS-Net), which was started in 2011 to promote standardization and publicity for 21 historical population databases from European and American countries and will eventually produce a major expansion in the spatial breadth of longitudinal data for Western historical populations. Such standardized EHPS-Net data will contribute not only a new understanding of the Western population in the past but also new comparisons between the West and the East. Basic information about this project is available on the EHPS-Net website (http://www.ehps-net.eu/).

2 According to available information online, these data have inspired some 10,000 scholarly publications, including more than 6,800 publications based on the IPUMS, 640 publications based on the BALSAC Population Database, 277 publications as of 2011 based on the Historical Sample of the Netherlands, 370 publications based on Le *Programme de Recherche en Démographie Historique*, 55 publications between 2005–2012 based on the Scanian Economic Demographic Database, 700 publications based on the Umea Demographic Database, and 1,700 publications based on the Utah Population Database.

3 These counts were produced by searches in Google Scholar on the names of the nine data sets that compose Fig. 1. We search separately for each five-year period, restricting results to those publications that include the full name of the data set.

4 In the case of IPUMS, perhaps many of these citations may refer to contemporary, not historical, IPUMS data.

5 Documenting these data to make them usable by researchers is similarly challenging. For recent examples of documentation, see Bourdieu et al. (2013) for an introduction to the latest such intergenerational longitudinal data, the French TRA, as well as Lee et al. (2010) and Wang et al. (2013) on the CMGPD-LN and CMGPD-SC.

6 A similar project comparing demographic behavior in historical populations in the Netherlands and Taiwan has yielded four volumes in the Life at the Extremes: The Demography of Europe and China (LatE) series (Chuang et al. 2006; Engelen and Hsieh 2007; Engelen and Wolf 2005; Engelen et al. 2012).

7 See Lee and Steckel (2006) and Goldstone (2011) for a discussion of the overall context and contribution of the Eurasian Project in Population and Family History to historical sociology and economic demography.

8 The Hong Kong University of Science and Technology School of Humanities and Social Science and the University of California, Los Angeles California Center for Population Research held three meetings in September 2010, August 2011, and June 2014 to bring together researchers working on historical household registers and genealogies in East Asia to facilitate coordination and comparisons. A parallel meeting organized by scholars at Seoul National University and Sungkyungkwan University in January 2012 provided additional opportunities for EAP II interaction and planning. A number of subsequent panels and presentations at the International Population Conference convened by the International Union for Scientific Study of Population, and annual meetings of the Population Association of America and the Social Science History Association reported EAP II findings and comparisons.

9 The only exception is the Historical Sample of the Netherlands, which by design is proportionally representative of the national population. See Mandemakers (2000) for the sampling design and other details of the HSN data. Even the well-known TRA historical data for France are not rigorously representative of France (Bourdieu et al. 2013).

10 See http://www.indepth-ishare.org/index.php/home for detailed information on the INDEPTH network data repository.

11 See http://www.icpsr.umich.edu/icpsrweb/ICPSR/series/265.

12 See http://ddmh.skku.edu/. These data were digitalized by a research group at Sungkyunkwan University, who offered the images and digitized data in 2003 on CD-ROM and recently online.

13 The Tansung register data distributed by researchers at Sungkyunkwan University were a series of cross-sections. James Lee, Cameron Campbell, Hao Dong, and their collaborators have linked these cross-sectional data to produce the longitudinal KMGPD-TS. These longitudinal links are available as a supplementary file named “Longitudinal Links to Construct the Korean Multi-Generational Panel Dataset-Tansung from the Tansung Household Registers” that can be merged into the original Tansung Household Registers files, and will be available for download at the Hong Kong University of Science and Technology Institutional Repository (repository.ust.hk/ir/).

14 See http://www.demography.sinica.edu.tw/EN/en_background.htm.

15 Population registers from other locations in Japan are still being entered, and some data sets created from already entered registers are being harmonized. Funding permitting, the Reitaku University Population and Family History Project hopes to make a consolidated data set more widely available at some time in the future. Interested parties should write to Satomi Kurosu (skurosu@reitaku-u.ac.jp), director of the Reitaku University Population and Family History Project, for more information.

16 Most of these data from other regions are constructed from household registers called *Shumon-aratame-cho* (SAC), which were more common in other regions. Although these two types of registers collected similar information, the original purpose of the SAC was to identify hidden Christians and prevent entry and spread of Christianity. The NAC were primarily for population registration and investigation (Tsuya and Kurosu 2004:290).

17 Ages in the CMGPD-LN, CMGPD-SC, KMGPD-TS, and NAC-SN are calculated by sui (in Chinese)/*sai* (in Japanese)/*se* (in Korean), a traditional way to calculate age in East Asia. A person is aged 1 *sui/sai/se* at birth and is one year older after each lunar new year. On average, an age measured in sui/sai/se is 1.5 greater than an age reckoned in the Western method. Because additional details about date of birth recorded in the registers appear unreliable, at present there is no means of directly calculating an age in Western years. To facilitate comparison with results from elsewhere in which ages are in Western years, we generally define age groups with the initial and final year offset by one year. For example, to produce something comparable with Western ages 5–9, we typically use the age range 6–10 *sui/sai/se*. Because the CTHRD was recorded in a continuous (person-event) format, it records only the individual’s birth date, not age. In this article, we calculate the ages in the CTHRD as current year – birth year + 1 to make them roughly comparable with ages recorded in Asian *sui/sai/se* in the other data sets.

18 In the case of the CTHRD these data are available only for Hsin-chu and Chu-shan.

19 See Plakans (1977) for discussion of the importance of linking noncoresident kin.

20 See the two user guides—Lee et al. (2010) and Wang et al. (2013)—for detailed introductions to the specific historical and institutional backgrounds of populations covered by the CMGPD-LN and CMGPD-SC. For additional relevant background, see Chen (2009), Ding et al. (2004), and Lee and Campbell (1997).

21 Unlike banner populations in other parts of China who were organized under the Office of the Eight Banner Command, the CMGPD-LN were mainly hereditary tenants on state land under the Imperial Household Agency (*neiwufu*), while the CMGPD-SC were under the Jilin Military Yamen, a specialized office in the General Office of the Eight Banner Command.

22 See Lee and Park (2008), Son (2007), and the edited volume by the Household Register Working Group (2003) for detailed introduction to the specific historical and institutional backgrounds of Korean household registers, especially for Tansung registers.

23 According to previous studies of the Korean registration system, not all households in the community were recorded in the registers in Tansung. Rather, the government sought to record a base population of sufficient size to support its operations in taxation and military, although such responsibility may actually be shared by all households in the community. See Kim et al. (2013) for a summary discussion on this issue as well as an examination of turnover in the recorded population.

24 *Nobi* is a complex category referring to different servile populations in Korea who are similar to yet different from Western serfs and slaves. There is considerable debate over its appropriate English translation. See Kim (2004) and Rhee and Yang (1999) for detailed discussion comparing Korean and Western servile populations.

25 See Hayami (1979) and Narimatsu (1985, 1992) for detailed introductions on the history and institutional settings of the Japanese household registers and these two villages.

26 Wolf and Huang (1980), in one of the first major studies to be based on these data, described them in some detail.

27 The complete CTHRD includes 22 research sites. However, data for only eight—An-ping, Bei-pu, Chui-ju, Chu-pei, E-mei, Lu-kang, Shen-gang, and Ta-chia–were made available for the analysis in this article. See Barclay (1954) and Katz and Chiu (2006) for specifics of the colonial Taiwan household registers.

28 Given that some individuals recorded in the CTHRD or their offspring may still be alive today, the data set does not include household addresses and individual names, criminal records, or records of opium smoking.

29 We could link the CMGPD-LN and CMGPD-SC manually because individuals—and more importantly, their villages and households—were mostly listed in similar order in successive registers. Because individuals were not listed in the same order in successive KMGPD-TS registers, we first identified by machine multiple candidate links based on matches of some or all individual characteristics, such as age, gender, and name. Our coders then reviewed these candidate links manually to select one.

30 For each individual recorded on a register page, coders first checked whether he/she already had an entry in the database by searching on name and birthdate. If a record already existed for that individual because he/she had appeared in the entry of another household, the new information was added to the existing record. Otherwise, a new record was created. The database later was then transcribed into a relational database, which consisted of several tables for different types of information that were connected by unique household and individual identifiers. See Wolf (2009) for specifics.

31 Because the CTRHD is organized differently, and records individuals on a continuous basis, this calculation is not relevant for it.

32 In addition to such common reasons for exit as out-marriage, out-migration, and death, the CMGPD-LN, CMGPD-SC, KMGPD-TS, and NAC-SN all include one particular administrative category of annotations: namely, escape (*tao* in Han Chinese, *do* in Korean, and *kakeochi* in Japanese). “Escape” refers to unauthorized out-migration of individuals from state purview and consequently from the household registers. See Campbell and Lee (2001) and Tsuya and Kurosu (2013) for comparisons between escape and other types of migration in the CMGPD-LN and NAC-SN respectively. See Dong et al. (2015) for a comparative analysis of escape behavior among the CMGPD-LN, KMGPD-TS, and NAC-SN, as well as household composition and social context determinants of such escapes.

33 The original CTHRD records events, not person-years. To produce comparable distributions for the CTHRD for Figs. 4, 5, and 6, we transformed the CTHRD data from person-event to person-year structure by adding annually repeated observations to individuals who are currently under observation in the data.

34 For the triennial CMGPD-LN and KMGPD-TS, estimates of the probability of dying in the next year are produced from the predicted probability of death in the next three years with the formula *p*_1_ = 1 – ((1 – *p*_3_)^1/3^).

35 See Shepherd (2001) for a study on smallpox and mortality based on the CTHRD data.

36 Table 4 in the appendix identifies and summarizes the characteristics and similarities of the EAP II data and other major longitudinal microdata sets.

37 Ruggles et al. (2011) described recent progress in longitudinal linking of historical censuses from the NAPP.
